# Measurement properties of the Inventory of Cognitive Bias in Medicine (ICBM)

**DOI:** 10.1186/1472-6947-8-20

**Published:** 2008-05-28

**Authors:** Ruth M Sladek, Paddy A Phillips, Malcolm J Bond

**Affiliations:** 1 School of Medicine, Flinders University, Adelaide, Australia; 2 Department of Medicine, Flinders Medical Centre, Noarlunga Hospital, Repatriation General Hospital, Flinders Drive, Bedford Park, Australia

## Abstract

**Background:**

Understanding how doctors think may inform both undergraduate and postgraduate medical education. Developing such an understanding requires valid and reliable measurement tools. We examined the measurement properties of the Inventory of Cognitive Bias in Medicine (ICBM), designed to tap this domain with specific reference to medicine, but with previously questionable measurement properties.

**Methods:**

First year postgraduate entry medical students at Flinders University, and trainees (postgraduate doctors in any specialty) and consultants (N = 348) based at two teaching hospitals in Adelaide, Australia, completed the ICBM and a questionnaire measuring thinking styles (Rational Experiential Inventory).

**Results:**

Questions with the lowest item-total correlation were deleted from the original 22 item ICBM, although the resultant 17 item scale only marginally improved internal consistency (Cronbach's α = 0.61 compared with 0.57). A factor analysis identified two scales, both achieving only α = 0.58. Construct validity was assessed by correlating Rational Experiential Inventory scores with the ICBM, with some positive correlations noted for students only, suggesting that those who are naïve to the knowledge base required to "successfully" respond to the ICBM may profit by a thinking style in tune with logical reasoning.

**Conclusion:**

The ICBM failed to demonstrate adequate content validity, internal consistency and construct validity. It is unlikely that improvements can be achieved without considered attention to both the audience for which it is designed and its item content. The latter may need to involve both removal of some items deemed to measure multiple biases and the addition of new items in the attempt to survey the range of biases that may compromise medical decision making.

## Background

The context of decision making in modern healthcare is complex, often involving multiple decision makers from varied professions. While their decisions may be imbedded in such broad organisational contexts, [1] doctors still retain a primary role in diagnostic and treatment decisions for patients, [2] ultimately determining which protocols to follow [3,4]. Optimising doctors' decision making is therefore a worthy objective. For example, if cognitive processes influence doctors' decision making, an improved understanding of these processes may contribute to maximising patient outcomes and avoiding common errors [5-8]. Our specific interest lies in understanding the cognitive processes that may inform strategies designed to change existing clinician practices so that gaps between the known best research evidence and existing clinical practices are reduced [9]. In sum, understanding how doctors think may contribute to both undergraduate and postgraduate medical education.

Such an understanding requires valid and reliable measurement tools. Currently available instruments include the Rational Experiential Inventory (REI) [10] and the Thinking Dispositions Composite [11] which measure general thinking styles. Even the Sensing-Intuiting and Thinking-Feeling subscales derived from the Myers-Briggs Type Indicator [12] have been proposed to measure cognitive style. To date relatively few scales have been designed to tap this domain with specific reference to medicine. The Inventory of Cognitive Bias in Medicine (ICBM) is one such potential instrument [13].

The ICBM was designed to measure the extent to which cognitive biases detract from logical and statistical thinking [13]. Items were constructed without reference to any specific theoretical model, although the authors drew on the substantial body of 'heuristics and biases' research from psychology, demonstrating that even experts fall victim to common biases in reasoning [14,15]. The ICBM comprises 22 items, each of which presents a clinical scenario to which responses represent either a 'correct' answer based on a statistical rationale or a 'bias prone' response [13]. That is, reasoning is assumed to be *either *rational (a statistically correct answer) *or *biased (a statistically incorrect answer).

The ICBM was originally administered to medical students, residents and physicians, with the students responding with a higher level of bias than the physicians. Later it was noted that students who attended a seminar on cognitive bias demonstrated significantly less biased responses than non-attendees [16]. Unfortunately, only modest internal reliability coefficients (α) of 0.62 for physicians and 0.42 for students/residents were obtained [13]. Our own research using the ICBM, as yet unpublished, also found a modest overall α (0.56), obtained from a similar sample of medical students (0.68), postgraduate trainee doctors (0.51) and medical consultants (0.41). The total sample figure was improved marginally by iteratively deleting items based on low item-total correlations until only 10 items remained (α = 0.61).

Clearly, despite apparent face validity and the critical importance of being able to assess medical decision making, the ICBM currently lacks both a theoretical basis and construct validity. Recently, over 30 years of heuristics and biases research was reconsidered in relation to emergent support for dual processing models of reasoning [17]. Such models propose two modes of cognitive processing; one referred to as experiential, heuristic, intuitive, unconscious and fast; the other as deliberate, reflective, rational, conscious and slow [18-21]. Within this framework a biased response to an ICBM item could be considered *prima facie *evidence for a heuristic mode of reasoning. While this potentially offers a theoretical basis for the ICBM, its construct validity can only be demonstrated by comparing responses with other test instruments that measure the same or similar constructs.

The current study had two objectives. First, we wished to examine the measurement properties of the ICBM, with the specific goal of improving internal consistency. Second, we sought to investigate the construct validity of the ICBM by comparing it with scores from the Rational Experiential Inventory (REI). Consistent with a dual processing model of reasoning, this instrument measures rational (need for cognition) and experiential (faith in intuition) thinking styles. It was hypothesised that if higher ICBM scores reflect rational reasoning and lower scores reflect experiential reasoning (cognitive bias), there would be positive associations with need for cognition and/or negative associations with faith in intuition.

## Methods

This study was undertaken in Adelaide, South Australia at Flinders Medical Centre (500 beds) and Repatriation General Hospital (280 beds), both metropolitan teaching hospitals affiliated with Flinders University. Ethics approval was obtained from the Research and Ethics Committees of both institutions.

### Participants and procedure

A study of thinking dispositions among doctors and medical students provided 147 participants. This was augmented by the addition of 201 participants. In total, 77 first year postgraduate entry medical students, 88 trainees (postgraduate doctors in any specialty) and 183 consultants were recruited to the study (N = 348). Each tranche of participants was recruited using a similar procedure. Questionnaires were mailed to doctors (emailed to students) with two follow-up reminders at two-week intervals. Due to initial low numbers of students, an additional invitation to participate was made personally during a teaching session.

### Measures

The data reported are age, gender, and scores for the ICBM and REI, respectively.

#### Inventory of Cognitive Bias in Medicine

The ICBM consists of 22 clinical scenarios, each of which is followed by a question with forced choice responses with either two or three alternatives, one of which represents a 'correct' answer based on a statistical rationale, while other alternatives represent 'bias prone' responses [13]. Scores reflect the total number of correct answers (ICBM22: 0–22). For example, item 8 describes a paediatrician whose last four patients have been girls. Participants are asked whether the next patient to be seen is likely to be a girl, a boy, or whether there is an equal chance of either. The statistically correct response is the last. The specific biases surveyed by the ICBM are summarised in Table 1.

**Table 1:** Representation of cognitive biases in the ICBM

**Bias**	**No. of Items**
Insensitivity to prior probability of outcomes	9
Use of irrelevant information in probability estimates	6
Insensitivity to sample size	5
Illusory correlation	5
Easily retrievable instances are judged to be more frequent	4
Framing of anchoring bias	2
Confirmatory bias	1
Misperceptions of chance	1
Insensitivity to the principle of regression	1
Insensitivity to superior reliability of objective over intuitive data	1

Note. Reprinted with permission from Academic Medicine [13].

#### Rational Experiential Inventory

The REI is a reliable and valid instrument containing scales that measure rational (need for cognition) and experiential (faith in intuition) thinking dispositions [10]. Need for cognition reflects the tendency to actively engage in, and enjoy, thinking. Faith in intuition measures the preference for experiential processing. Within each scale an ability subscale assesses how well a person believes they use each disposition, while a favourability subscale assesses reliance on and enjoyment of each disposition. There are 40 questions with 5-point response scales (20 each for need for cognition and faith in intuition, with 10 items each for the subscales of ability and favourability). Scores are averaged to provide variables ranging from 1 to 5, with a higher score reflecting a greater tendency to endorse the construct measured. The current sample provided internal reliabilities (α) of 0.90 (total need for cognition), 0.81 (need for cognition: ability), 0.82 (need for cognition: favourability), 0.79 (total faith in intuition), 0.74 (faith in intuition: ability), 0.63 (faith in intuition: favourability).

## Results

Table 2 presents the number of correct responses to individual ICBM items, and indicates any group differences in correct responding. An indication of linear trend (students, trainees, consultants) in correct responses is also provided. Item names are based on the key content of each scenario. Table 3 summarises all relevant study variables. Probability values for both absolute group difference and linear trend are again included. While the total sample comprised approximately 61% males, they were differentially distributed across the student, trainee and consultant subgroups. Predictably, age increased from students to trainees to consultants. Need for cognition provided subtle and varying results across the subsamples, while there was a clear trend toward a decrease in faith in intuition with experience. Correct responses to both the ICBM22 and a 10 item ICBM (ICBM10), based on findings from an unpublished data (items 2, 4, 5, 6, 7, 10, 13, 14, 15, 17), increased with experience.

**Table 2:** Correct responses to ICBM items.

	**Students**	**Trainees**	**Consultants**		
	**n (%)**	**n (%)**	**n (%)**	**p**_difference_	**p**_trend_

1. Training program	19 (24.7)	26 (29.5)	59 (32.4)	.459	.217
2. Atrial fibrillation	37 (48.1)	73 (83.9)	132 (72.9)	< .001	.001
3. Malpractice	50 (64.9)	62 (71.3)	154 (84.6)	.001	< .001
4. Effeminate	11 (14.3)	20 (23.0)	68 (37.6)	< .001	< .001
5. Single mother	29 (37.7)	25 (28.7)	57 (31.3)	.450	.420
6. Leg pain	25 (32.5)	45 (51.7)	104 (57.8)	.001	< .001
7. Reflux	34 (44.2)	66 (75.0)	117 (64.3)	< .001	.018
8. Paediatrician	71 (92.2)	83 (94.3)	164 (90.6)	.575	.530
9. Hypothyroid	60 (77.9)	52 (59.8)	104 (57.1)	.006	.003
10. Breast nodule	58 (75.3)	65 (74.7)	159 (87.4)	.013	.008
11. Lung cancer	68 (88.3)	74 (85.1)	164 (90.6)	.403	.431
12. Phenylketonuria	3 (3.9)	5 (5.7)	13 (7.2)	.590	.306
13. Diabetes	40 (51.9)	69 (78.4)	138 (75.8)	< .001	.001
14. Female CAD	22 (28.6)	30 (34.1)	85 (46.7)	.012	.004
15. Joints	14 (18.2)	19 (21.8)	55 (30.2)	.085	.030
16. Angina CHD	26 (33.8)	33 (37.5)	68 (37.4)	.842	.623
17. Professor	36 (46.8)	46 (52.3)	103 (56.6)	.340	.143
18. Information source	21 (27.3)	17 (19.3)	33 (18.0)	.231	.114
19. Hollywood death	65 (84.4)	85 (97.7)	177 (96.7)	< .001	.001
20. Birth rates	32 (41.6)	38 (43.7)	70 (38.3)	.676	.520
21. Blood pressure	51 (66.2)	51 (58.6)	130 (71.0)	.127	.253
22. Twin weights	9 (11.7)	8 (9.2)	24 (13.2)	.638	.600

**Table 3:** Summary statistics for key study variables.

	**Students**	**Trainees**	**Consultants**	**p**_difference_	**p**_trend_
Gender (n, % male)	30 (39.0)	47 (53.4)	136 (74.3)	< .001	< .001
Age in years (mean, SD)	26.4 (5.7)	32.5 (6.9)	47.7 (9.8)	< .001	< .001
REI (mean, SD)					
Need for cognition (total)	4.0 (0.4)	3.9 (0.4)	3.9 (0.4)	.054	.027
Need for cognition (ability)	4.1 (0.5)	3.8 (0.6)	4.0 (0.4)	.018	.203
Need for cognition (favourability)	4.0 (0.4)	3.9 (0.4)	3.8 (0.4)	.020	.009
Faith in intuition (total)	3.3 (0.5)	3.2 (0.6)	3.0 (0.5)	< .001	< .001
Faith in intuition (ability)	3.5 (0.5)	3.4 (0.5)	3.3 (0.5)	.006	.002
Faith in intuition (favourability)	3.2 (0.7)	3.1 (0.7)	2.8 (0.6)	< .001	< .001
ICBM22 (mean, SD)	10.1 (2.9)	11.4 (2.8)	12.0 (2.9)	< .001	< .001
ICBM10 (mean, SD)	4.0 (2.1)	5.3 (2.0)	5.6 (2.0)	< .001	< .001
ICBM17 (mean, SD)	7.3 (2.7)	8.7 (2.6)	9.1 (2.8)	< .001	< .001
ICBMs1 (mean, SD)	4.4 (2.1)	4.6 (2.3)	5.2 (2.4)	.016	.007
ICBMs2 (mean, SD)	4.7 (2.1)	6.2 (1.8)	6.6 (1.9)	< .001	< .001

The internal reliability (α) of the ICBM22 was 0.57 (0.55 students, 0.52 trainees, 0.56 consultants), indicating a relatively poor level of internal consistency. The ICBM10 demonstrated similarly poor reliability of 0.57 (0.55 students, 0.54 trainees, 0.52 consultants). In light of these internal consistency figures, two strategies were used in the attempt to improve this property of the ICBM.

### Item-total Correlation Analysis

First, iterative removal of those items with the lowest item-total correlation was undertaken, identical to the procedure previously used to derive the ICBM10. With the current data, α was maximised with a 17 item scale (ICBM17). Items 8, 11, 18, 21 and 22 were omitted. These items assessed misconceptions of chance (8), framing or anchoring bias (11 and 22), insensitivity to superior reliability of objective over subjective data (18), and insensitivity to the principle of regression (21). The resultant 17 item scale achieved an α of 0.61 (0.56 students, 0.57 trainees, 0.61 consultants), with item-total correlations ranging from 0.11 to 0.37.

Removal of a further item resulted in the total sample α being maintained at 0.61 but a reduction in consistency for two of the three subsamples (0.58 students, 0.55 trainees, 0.59 consultants). This result suggested that further gains in internal consistency could only be attained through differential item removal for each subsample. That is, different scales for different target groups. Summary statistics for the ICBM17 are included in Table 3. As with the ICBM22, correct responses increased with experience.

### Factor analysis

Second, factor analysis was used as a pragmatic guide to potential subscale membership among the ICBM items [22]. Decisions regarding the nature of the factor analysis were based on this strategy. Maximum likelihood extraction was used to allow generalisation from a sample to a population [23] and for correlations with more unique variance and less error variance to be given more weight [22]. Most importantly, adjustment is made for the constraints imposed on the data based on the increased potential for non-random measurement error associated with dichotomous variables.

This procedure resulted in an initial solution comprising 10 factors, each with an eigenvalue greater than one, although only 60% of the variance among items was accounted for by these factors. This result reflects the tendency for dichotomous variables to cluster together due to similar response distributions rather than actual item content [24], producing additional factors that are essentially statistical artefacts. Therefore, a conservative decision rule for retaining factors for rotation was employed. Parallel analysis criteria [25] takes account of both sample size and the number of items being analysed and is more reliable than the misunderstood 'eigenvalues greater than one' rule [26], particularly when there are many coefficients of modest size within the available correlation matrix (communality range 0.025 to 0.197). Further, an oblique rotation of the retained factors was undertaken using the oblimin criterion (delta = 0), to acknowledge the expected intercorrelations among any dimensions of the ICBM.

Using this strategy only two factors were retained for rotation, accounting for a mere 19% of variance. Scales were nevertheless computed from these factors. Scale 1 (ICBMs1) comprised items predominantly concerned with insensitivity to sample size and representativeness (items 1, 4, 5, 6, 9, 10, 12, 14, 15, 16, 17, 20). Scale 2 (ICBMs2) tended to tap availability and representativeness (items 2, 3, 4, 6, 7, 10, 12, 13, 14, 15, 19). The substantial overlap in the content of these two scales should be noted (*r *= .68, *p *< .001). Interestingly, the five items that did not load on either or both of these scales were identical to those excluded by the item-total correlation analysis. Summary statistics for these scales are included in Table 3. For both scales, correct responses increased with experience. Internal consistency was 0.58 (0.52 students, 0.56 trainees, 0.61 consultants) for ICBMs1 and 0.58 (0.60 students, 0.47 trainees, 0.51 consultants) for ICBMs2.

Correlations between the various versions of the ICBM that we have presented, and scores from the REI, are shown in Table 4 separately for students, trainees and consultants. Among students there are a number of significant, albeit modest, coefficients. In accord with theory, all ICBM versions are negatively correlated with faith in intuition, although this is accounted for by favourability rather than ability. There is also a less consistent positive association between ICBM and need for cognition among students. This is reflected most by the ICBM22, ICBM17 and ICBMs2 (availability/representativeness). Among trainees significant correlations are more patchy, although again the direction of associations is in accord with theory. These data offer little evidence to support one version of the ICBM over another. Similarly there is no evidence available from the consultant data to either guide the choice of ICBM version or support the assumption that the ICBM taps a related construct to that operationalised by the REI.

**Table 4:** Correlations between ICBM scores and the Rational Experiential Inventory.

	**Rational Experiential Inventory**					
	
	**Need for Cognition**			**Faith in Intuition**		
		
	**Total**	**Ability**	**Favourability**	**Total**	**Ability**	**Favourability**
Students						
ICBM22	.28*	.24*	.26*	-.26*	-.10	-.33**
ICBM10	.20	.17	.19	-.29**	-.16	-.34**
ICBM17	.29**	.24*	.28*	-.28*	-.13	-.35**
ICBMs1	.19	.14	.21	-.25*	-.18	-.27*
ICBMs2	.22*	.18	.23*	-.18	-.01	-.28*
Trainees						
ICBM22	.13	.13	.08	-.18	-.22*	-.12
ICBM10	.23*	.27*	.10	-.13	-.16	-.08
ICBM17	.15	.16	.08	-.18	-.24*	-.10
ICBMs1	.14	.12	.11	-.10	-.17	-.03
ICBMs2	.15	.21*	.02	-.15	-.18	-.11
Consultants						
ICBM22	.06	.05	.05	-.11	-.09	-.11
ICBM10	.08	.07	.06	-.08	-.07	-.07
ICBM17	.06	.05	.05	-.09	-.08	-.07
ICBMs1	.02	.03	.01	-.04	-.05	-.03
ICBMs2	.12	.09	.13	-.08	-.04	-.09

## Discussion

To gain acceptance as a useful measurement instrument there are a number of characteristics that any test should demonstrate. Three such characteristics have been reported in the current study. First, the test should possess content validity. That is, it should contain items that appropriately sample the construct to be measured. Second, there should be internal consistency among the chosen items. That is, it is an expectation that items purporting to measure the same construct should demonstrate reasonable item-total correlations before they are used to create a summative scale. This is commonly examined using α. Third, the instrument should demonstrate a reasonable level of construct validity. That is, different measurement tools measuring constructs from similar domains should exhibit relationships that are in accord with underlying theory.

The data presented suggest that the ICBM potentially fails all of these examinations. Considering the latter first, only modest evidence was available of theoretically-supported links between the ICBM and scores from the REI. Such evidence was best illustrated by presenting separate coefficients of association for each of the subsamples. These relationships could generously be described as encouraging for the student group, tenuous among trainees, and non-existent for consultants. Admittedly, construct validity for the ICBM could only be examined using the REI. It is possible that other instruments from the thinking dispositions domain may offer more encouraging results. Nevertheless, we have found in a series of studies [27,28], some as yet unpublished, that the REI is reliable, valid and predictive, and as such is an entirely sensible standard with which to compare the ICBM. More probable is that the poor associations between the ICBM and REI are artefacts, stemming from the similarly poor internal reliability coefficients that we have presented. No coefficient achieved the level of internal consistency normally considered acceptable for mature scales (.70), although most reached the level deemed respectable for scales under development (.50–.60) [29].

Failure to attain more appropriate internal consistency draws attention to the very content of the ICBM. Interestingly, the original evidence supporting the validity of the ICBM merely comprised a content review by experts [13]. Yet there remains inherent uncertainty regarding exactly which heuristics or biases are assessed by each ICBM item. The authors [13] identified 35 biases that were addressed by the 22 items (Table 1), without specific direction as to which item tapped which bias(es). Clearly some items tap more than one bias, there is selective bias (*sic*) among the biases chosen for measurement, and also significant overlap across some biases concerning the logical error committed by choosing the incorrect response. Unfortunately these observations mean that it remains unknown whether items address "heuristic reasoning", "rational reasoning", or indeed both.

The above comments help to explain the pattern of results obtained from both the item-total correlation analysis and the factor analysis. The five items removed during the former procedure were extrapolated by us as tapping relatively unique biases assessed by only one or two items (misconceptions of chance, framing/anchoring, insensitivity to superior reliability of objective over subjective data, insensitivity to the principle of regression). These five items were equally ineffective in demonstrating shared variance in the factor analysis. The two factors identified accounted for little variance themselves, and there was significant overlap of item content (insensitivity to sample size/representativeness, availability/representativeness). On the one hand this result may reflect the lack of one-to-one measurement of biases among some items, as noted above, while on the other hand perhaps it simply mirrors the biases that are most frequently assessed among the ICBM items.

Rather than measure a thinking style or styles *per se*, our results also suggest that it is more likely that the ICBM in fact taps a relevant knowledge base that increases with professional experience. While all other results are equivocal, there is clear evidence (Table 3) that correct responses to all versions of the ICBM presented increase significantly with medical experience. Interestingly, and perhaps most importantly, correlations between the ICBM and REI for students (Table 4) further suggest that those who are naïve to the knowledge base required to "successfully" respond to the ICBM may nevertheless profit by a thinking style in tune with logical reasoning (i.e., relatively high need for cognition and/or relatively low faith in intuition). This appears particularly true for those naïve participants who expressed favouring such a thinking style. These observations are in accord with one of the original propositions underlying the development of the ICBM, which suggested its use as a potential teaching tool [13].

## Conclusion

It is unlikely that improvement of the ICBM can be achieved without considered attention to both the audience for which it is designed and a careful analysis and revision of the items themselves. The latter may need to involve both removal of some scenarios deemed to measure multiple biases and the addition of new items in the attempt to more appropriately and fulsomely survey the range of biases now understood to compromise decision making in the medical domain. Such efforts, while substantial, represent the logical prerequisite to the establishment of content validity for the ICBM. Nevertheless, such efforts may yet prove fruitful given the contemporary interest on the role of cognitions in medical decision making.

## Abbreviations

ICBM: Inventory of Cognitive Bias in Medicine; ICBM22: Original 22 question version [13]; ICBM10: 10 question-version found by authors in an earlier study; ICBM17: 17 question-version based on iterative removal of those items with the lowest item-total correlation; ICBMs1: Scale 1 identified through factor analysis; ICBMs2: Scale 2 identified through factor analysis; REI: Rational Experiential Inventory.

## Competing interests

The authors declare that they have no competing interests.

## Authors' contributions

RMS, PAP and MJB conceived and designed this study. RMS undertook all data acquisition. RMS and MJB undertook the primary data analysis and interpretation, while MJB conducted the specialised analyses. RMS and MJB drafted the paper. All authors were involved in its critical revision and final approval for publication.

## Pre-publication history

The pre-publication history for this paper can be accessed here:


